# Human umbilical cord mesenchymal stem cells promote steroid-induced osteonecrosis of the femoral head repair by improving microvascular endothelial cell function

**DOI:** 10.18632/aging.205794

**Published:** 2024-04-29

**Authors:** Junwen Chen, Wenyi Jin, Changheng Zhong, Wenxiang Cai, Liangkun Huang, Jianlin Zhou, Hao Peng

**Affiliations:** 1 Department of Orthopedics, Renmin Hospital of Wuhan University, Wuhan 430062, China; 2 Department of Biomedical Sciences, City University of Hong Kong, Kowloon Tong, Hong Kong 999077, Hong Kong Special Administrative Region

**Keywords:** human umbilical cord mesenchymal stem cells, osteonecrosis, bone microvascular endothelial cells, core decompression

## Abstract

Recently, there has been growing interest in using cell therapy through core decompression (CD) to treat osteonecrosis of the femoral head (ONFH). Our study aimed to investigate the effectiveness and mechanism of human umbilical cord mesenchymal stem cells (hUCMSCs) in treating steroid-induced ONFH. We constructed a steroid-induced ONFH rabbit model as well as dexamethasone (Dex)-treated bone microvascular endothelial cells (BMECs) model of human femoral head. We injected hUCMSCs into the rabbit femoral head via CD. The effects of hUCMSCs on steroid-induced ONFH rabbit model and Dex-treated BMECs were evaluated via micro-CT, microangiography, histology, immunohistochemistry, wound healing, tube formation, and western blotting assay. Furthermore, we conducted single-cell RNA sequencing (scRNA-seq) to examine the characteristics of endothelial cells, the activation of signaling pathways, and inter-cellular communication in ONFH. Our data reveal that hUCMSCs improved the femoral head microstructure and bone repair and promoted angiogenesis in the steroid-induced ONFH rabbit model. Importantly, hUCMSCs improved the migration ability and angioplasty of Dex-treated BMECs by secreting COL6A2 to activate FAK/PI3K/AKT signaling pathway via integrin α1β1.

## INTRODUCTION

Osteonecrosis of the femoral head (ONFH) is defined by the localized death of osteocytes and bone marrow components due to reduced or interrupted blood supply to the femoral head. In this disorder, the balance between bone formation and bone destruction is disrupted, ultimately leading to structural collapse and destruction of the femoral head, causing pain and dysfunction of the hip joint in patients. In China, the cumulative number of patients with non-trauma-induced femoral head necrosis has reached 8,120,000, with glucocorticoids, alcohol, and hyperlipidemia as the main risk factors [1]. Many scholars have proposed hypotheses for the pathogenesis of the steroid-induced ONFH, including apoptosis of osteoblasts, and the disruption of bone homeostasis [2], among others. The role of bone microvascular endothelial cells (BMECs) in steroid-induced ONFH was previously emphasized, and the proposed “angiogenic-osteogenic coupling” [3, 4] further underscores the pivotal role of angiogenesis in repairing osteonecrosis. In addition, glucocorticoids directly affect BMECs, leading to their apoptosis, dysfunction, and the development of a hypercoagulable state [5]. Additionally, BMECs are arranged in the inner layer of blood vessels and play an extremely critical role in bone repair angiogenesis. Moreover, the dysfunction of BMECs has an essential role in the occurrence of steroid-induced ONFH [6]. Under this background, the present study explores how to protect and rescue the functions of BMECs.

Mesenchymal stem cell (MSC) therapy has shown promising applications in several fields, mainly including ischemic cardiomyopathy [7], acute respiratory distress syndrome [8], autoimmune diseases [9], and osteoarthritis [10]. Compared with other types of MSCs, human umbilical cord mesenchymal stem cells (hUCMSCs) have gained attention in cell therapy due to their advantages of being accessible by non-traumatic means, low immunogenicity, high *in vitro* expansion capacity, and ethical compliance [11]. Additionally, hUCMSCs have demonstrated powerful repair capabilities in repairing diseases, such as kidney injury [12] and skin injury [13]. Scholars have treated ONFH via the arterial injection of hUCMSCs, and the results demonstrated a marked reduction in the volume of ONFH in patients without serious complications [14]. However, the underlying mechanism of action remains unclear. This mechanism of action may be related to the regulation of the local immune environment by hUCMSCs [15]. The administration routes of cell therapy for ONFH mainly include arterial and local injections. Currently, core decompression (CD) combined with stem cell injection is the most mainstream operative approach [16]. In this treatment, stem cells are injected directly into the affected area while reducing intraosseous pressure and scraping away dead bone tissue, avoiding problems of stem cell homing.

Overall, hUCMSCs-based cell therapy combined with CD has vital clinical translational value. This study aimed to inject hUCMSCs directly into the femoral head via CD and systematically evaluate the therapeutic effects of hUCMSCs on a methylprednisolone (MPS)-induced ONFH rabbit model using micro-CT, angiography, histology, and tissue immunochemistry. The potential mechanism of action of hUCMSCs in ONFH was explored by using a scRNA-seq database and validated *in vitro* by studying Dex-treated BMECs of human femoral head.

## MATERIALS AND METHODS

### *In vivo* animal experiments

The experiments were performed at the Animal Experiment Center of the Renmin Hospital, Wuhan University. All animal procedures followed the guidelines for laboratory animal care and adhered to the ARRIVE guidelines. Ethical approval for the experiments was obtained from the Laboratory Animal Ethical and Welfare Committee of the Renmin Hospital of Wuhan University (WDRM20220106). Thirty-six male New Zealand rabbits weighing 3 ± 0.5 kg were procured from the Animal Experiment Center of the Renmin Hospital, Wuhan University.

On the first day of the experiment, an intravenous injection of 10 μg/kg lipopolysaccharide (LPS) (Sigma, USA) was administered along the marginal ear vein, followed by intramuscular administration of 20 mg/kg/day MPS (Pfizer, USA) on days 2 to 4. The rabbits were randomly divided into three groups, with 12 rabbits in each group: 1. the MPS group; 2. the CD group; 3. the CD+hUCMSC group.

#### Animal surgery

The rabbits were subjected to anesthesia using 3% pentobarbital sodium at a dosage of 30 mg/kg, supplemented with inhalation anesthesia for assistance. The operation was performed in an animal fluoroscopy operating room. In brief, a 1 mm Kirschner wire was drilled along the center of the femoral neck under fluoroscopy of the C-arm machine (Supplementary Figure 1A), a 2 mm hollow drill was drilled along the Kirschner wire to 3 mm below the cartilage (Supplementary Figure 1B). In the CD+hUCMSCs group, 0.2 ml of pluronic F127 hydrogel (sigma, USA) containing hUCMSCs was slowly injected (hUCMSCs concentration of 5 × 10^6^ cells/ml). In the CD group, a syringe was used to inject 0.2 ml of pluronic F127 hydrogel. The pluronic F127 hydrogel was prepared with reference to a previous study [17]. In the MPS group, rabbits were anesthetized, and the femur was exposed by making an incision in the skin, which was then sutured.

The wounds were closed using non-absorbable sutures, and a single intramuscular injection of 400,000 units of penicillin was administered after the surgery. The rabbit femur specimens were collected after eight weeks.

#### Microangiography of the femoral head

Five rabbits were taken from each group for the operation according to the previously reported method and reagent supplier guidelines [18]. Following the induction of anesthesia, Heparin saline (20 U/ml) was instilled through a disposable infusion device, and 4% paraformaldehyde was instilled. Finally, Microfil (Microfil MV-122, Flow Tech, USA) was instilled via syringe at 150 ml according to the reagent supplier’s protocol. It was noted that the same instillation rate was maintained for each animal as closely as possible. The animals were then executed via injection with an overdose of 3% sodium pentobarbital and left overnight in the refrigerator at 4°C. Samples of the proximal femur were removed and fixed in 4% paraformaldehyde for 48 h, followed by decalcification with ethylenediaminetetraacetic acid (EDTA, 10%, pH 7.4).

#### Micro-CT

The femoral specimen was secured in a sample tube and subjected to scanning using a micro-CT (SkyScan 1276 micro-CT system, Bruker, Kontich, Belgium). The parameters were set as follows: peak energy, U = 85 kV, I = 200 μA. The scanning slice gap was 10.5 μm, and the intermediate frequency resolution was 1024 × 1024. The data were acquired and reconstructed with the NRecon software (v1.7.4.2), and the 3D reconstruction was carried out using CTvox (v3.3). Subsequently, measurements of bone volume/total volume (BV/TV, %), trabecular number (Tb.N, 1/mm), trabecular separation (Tb.Sp, μm), and trabecular thickness (Tb.Th, μm) were performed using CTAn (v1.17.7.2) and CTvol (v2.3.2).

#### Histological analysis of the femoral head

To prepare for observation, the proximal femur samples were first fixed in 4% paraformaldehyde and then decalcified using 10% EDTA. The samples were dehydrated and embedded in paraffin. From the paraffin-embedded samples, we prepared sections 4 μm in thickness. These sections were subjected to Hematoxylin and eosin (HE) staining and viewed under a light microscope. To compute the cavity ratio in the bones, three fields of view were randomly selected.

For immunohistochemistry, the sections were incubated overnight at 4°C with a CD31 primary antibody (1:800, Novus Biologicals, USA). Subsequently, after incubation with a secondary antibody, the sections were visualized and photographed under a light microscope (Olympus, Japan). The counting of microvessels was conducted independently by two researchers.

### Single-cell profiling

#### Data of single cell source collection and analysis

The scRNA-seq data of human normal femoral head and ONFH were obtained from SRP361778 via the SRA database (https://www.ncbi.nlm.nih.gov/sra/). To identify the top 2000 most variable genes, a normalized expression matrix was utilized. Next, principal components analysis (PCA) was used for dimensionality reduction, which helped eliminate batch effects using the top 50 PCA components. In the clustering analysis, a clustering algorithm was utilized to visualize the data through techniques such as uniform manifold approximation, projection (UMAP), and t-distributed stochastic neighbor embedding (t-SNE).

In the analysis, we utilized the “FindAllMarkers” function with specific parameter settings (logfc.threshold = 0.25, min.pct = 0.25, and min.diff.pct = 0.25) to identify marker genes for each cluster. The first 100 marker genes were subjected to a gene ontology (GO) enrichment analysis to gain insights into their functional annotations. Additionally, cell annotation was performed based on previous knowledge of cell marker genes combined with GO analysis.

#### Pathway enrichment analysis

To further investigate the differentially expressed genes (DEGs) within cell families, we conducted GO and Kyoto Encyclopedia of Genes and Genomes (KEGG) enrichment analysis using the clusterProfiler R package (adjusted *p*-value < 0.05).

#### Analysis of intercellular communication

To explore the potential mechanisms underlying the interaction between stem cells and ECs, we employed the CellChat package (version 1.4.0) to analyze intercellular communication between different cell types (set *p*-value < 0.05).

### *In vitro* experiments

#### Isolation, culture, and identification of BMEC

The study was conducted in accordance with the Declaration of Helsinki, and approved by the Ethics Committee of Wuhan University (WDRM2021-KS068). In this study, we collected tissue samples from the femoral head of one individual donor (male, 48 years old) who underwent total hip arthroplasty for coxitis secondary to developmental dysplasia of the hip (DDH), and we collected operative residual tissue samples after obtaining informed consent from the donor.

Operating according to the previously reported method [19], we treated the bone with DMEM medium containing 2% type I collagenase (Servicebio, China) and 0.25% EDTA (Servicebio, China). The oil and fine bone particles were filtered out using a 70 μm cell sieve (Biosharp, China), and the cell suspension was collected. Next, the cell suspension was centrifuged, and the supernatant was removed. The cells were cultured using ECM (ScienCell, USA) medium.

Following a 24 h incubation period, the medium was removed. The cell culture dishes were then gently rinsed with pre-warmed HBSS to remove any unadhered cells. This washing step was repeated thrice. Subsequently, a fresh culture medium was added to the dishes, and the culture was continued. When the cells grew to 80–90% of the culture dish, the cells were collected using 0.25% EDTA, trypsin concentration of 0.1%, and re-inoculated culture in a 1:2 ratio. After two passages, we prepared for subsequent experiments.

To identify the primary cells, we used immunofluorescence. First, we fixed the cells using 4% paraformaldehyde for a duration of 10 minutes. Then, the cells were permeabilized with a 0.2% Triton X-100 PBS solution and incubated in a PBS solution containing 1% BSA for 30 minutes. Next, the primary antibodies VWF (1:400, Servicebio, China) and CD31 (1:200, Servicebio, China) were added to the cells and incubated overnight at 4°C. Subsequently, the cells were incubated with a fluorescent secondary antibody for an hour at room temperature, away from light. Finally, the cells were visualized and photographed under a fluorescent microscope (Olympus, Japan).

#### Cell culture

The hUCMSCs were purchased from Shenzhen Wingor Biotechnology Co., Ltd [20]. Moreover, the cells used in the experiments were obtained from 3-6 generations of the same batch of cells. The cells were cultured using the DMEM/F12 (HyClone, USA) supplemented with 10% FBS (Serapro, USA) and a 1% penicillin mixture (Biosharp, China). The cell culture was maintained at 37°C with 5% CO_2_. The medium was refreshed every 2–3 days when the cells reached approximately 80–90% passage.

BMECs were divided into three groups: the Con group; the Dex group; the Dex+hUCMSC group. Cell co-culture was performed in a non-contact co-culture manner. In 6, 24, and 96-well plate Transwell co-culture systems (0.4 μm, Corning, USA), hUCMSCs were inoculated in the upper chamber and BMECs in the lower chamber in a 2:1 cell ratio and cultured with ECM.

#### Cell counting Kit- 8 (CCK-8)

To determine the viability of BMECs, the CCK-8 (Beyotime Institute of Biotechnology, China) assay was performed following the supplier’s instructions. BMECs were incubated with 5000 cells per well in 96-well plates and treated with varied concentrations of Dex (50, 100, 150, 200, and 300 μM) for a duration of 24 h. After the treatment period, CCK-8 (10 μl) solution was added to each well and incubated for 2 h at 37°C in 5% CO_2_. The absorbance at 450 nm was then measured using a microplate reader (Perkin Elmer, USA).

#### Enzyme-linked immunosorbent assay (ELISA)

The concentration of COL6A2 was measured using an ELISA kit (BYabscience, China) according to the instructions provided by the manufacturer.

#### siRNA transfection

For the transformation of hUCMSCs, the RNAiMAX reagent was used following the manufacturer’s instructions. The cells were treated with negative control (NC) RNA and COL6A2 siRNA (RIBOBIO), respectively.

#### Wound healing assay

The BMECs were grown in 6-well plates until they reached 90–100% coverage. Then, they were co-cultured with either Dex-treated cells or hUCMSCs for 24 h. To create scratches on the cells, a 200 μl pipette tip was used. The scratches were visually examined and photographed. The ImageJ Software (version 3.0) was utilized to analyze the data at 0 and 24 h. The percentage of scratch shortening was calculated using this software.

#### Tube formation assay

A tube-forming assay was employed to evaluate the angiogenic capacity of BMECs *in vitro*. In pre-cooled 24-well plates, 250 μl of matrix gel (Corning, USA) was added and incubated at 37°C for 30 minutes. BMECs were then inoculated in ECM without FBS and incubated at 37°C in 5% CO_2_ for 6 h. The resulting tube formations were observed, photographed, and analyzed using the ImageJ Software.

#### Western blot analysis

The total protein from each group of BMECs was derived using RIPA lysis buffer, and the total protein was measured using a BCA kit (Servicebio, China). After electrophoresis and electrotransfer, the membranes were incubated with primary antibodies (p-FAK, FAK, p-PI3K, PI3K, p-AKT, AKT at a dilution of 1:1000 from Cell Signaling Technology, USA; GAPDH from Servicebio, China) overnight at 4°C. Subsequently, the membranes were incubated with secondary antibodies for 1 hour at 37°C. The levels of protein quantification were visualized using the ImageJ Software.

### Statistical analysis

The SPSS software (v22.0) was used for data analysis. Data were presented as mean ± standard deviation. For normally distributed data, *t*-tests, one-way ANOVA, and Tukey’s test were applied. *P*-value < 0.05 was considered statistically significant. All experiments were conducted in triplicate.

### Data availability statement

The data presented in this study are available on request from the corresponding author.

## RESULTS

### Results of *in vivo* research

#### hUCMSCs improved the femoral head microstructure in the MPS-induced ONFH rabbit model

**Figure 1:**
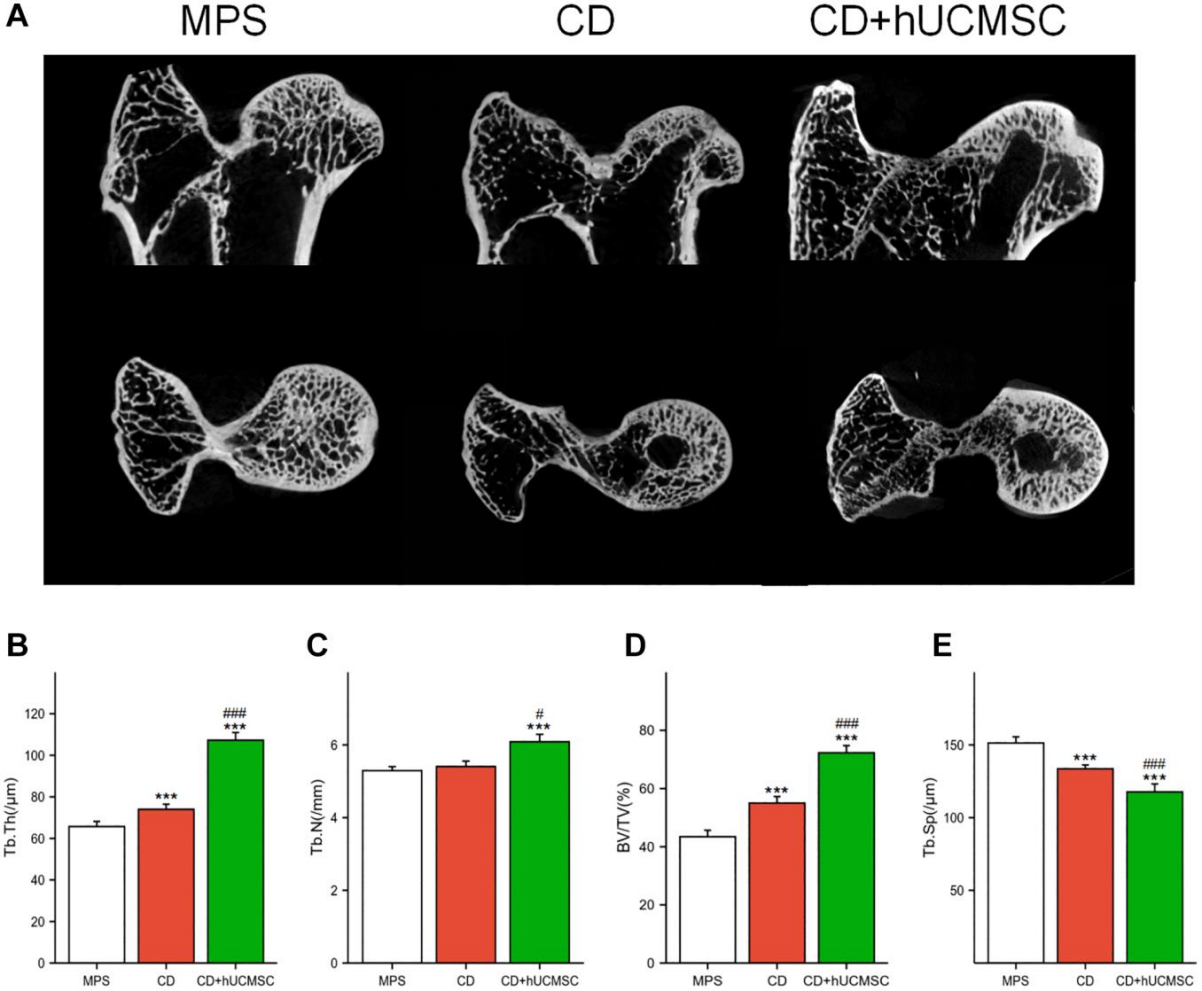
**hUCMSCs improved the femoral head microstructure in the MPS-induced ONFH rabbit model.** (**A**) Representative images of Micro-CT of the femoral head in each group; (**B**–**E**) Quantitative analysis of Tb.Th, Tb.N, BV/TV and Tb.Sp in each group. The data are presented as the means ± SD (*n* = 7). ^*^p < 0.05, ^**^p < 0.01, ^***^p < 0.001, compared with the MPS group. ^#^*p* < 0.05, ^##^*p* < 0.01, ^###^*p* < 0.001, compared with the Dex group.

Micro-CT was employed for the quantitative analysis of the microstructure of the femoral head. In the MPS rabbit femoral head, the trabeculae were observed to be sparse and disorganized. However, in the CD+hUCMSCs group, the trabeculae appeared to be complete and well-aligned (Figure 1A). Furthermore, when comparing the CD+hUCMSCs group with the MPS group, significant differences were observed in several parameters. The CD+hUCMSCs group exhibited significantly higher values for Tb.Th, Tb.N and BV/TV. Additionally, the CD+hUCMSC group displayed a significantly lower value for Tb.Sp compared with the MPS group. The implantation of hUCMSCs inhibited the occurrence of osteonecrosis and promoted bone regeneration in the femoral head of the ONFH rabbit model, thus improving the aforementioned microstructure of the femoral head (Figure 1B–1E).

#### hUCMSCs promoted angiogenesis in the femoral head in the MPS-induced ONFH rabbit model

**Figure 2:**
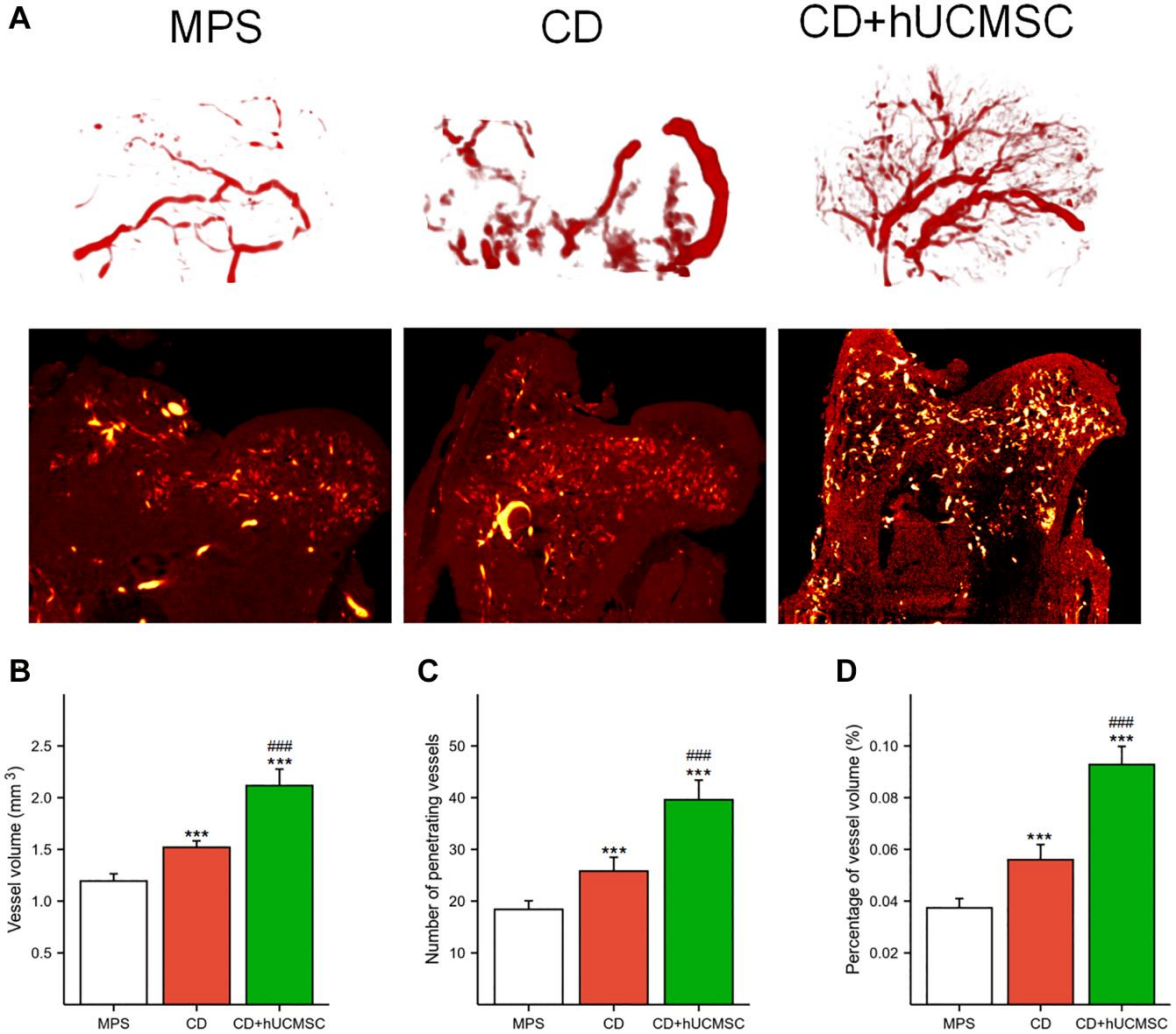
**hUCMSCs promoted MPS-induced angiogenesis in the femoral head in a rabbit model of ONFH.** (**A**) Representative microvascular imaging of the femoral head in each group. (**B**–**D**) Quantitative analysis of the vessel volume, number of penetrating vessels and percentage of vessel volume. The data are presented as the means ± SD (*n* = 5). ^*^p < 0.05, ^**^p < 0.01, ^***^p < 0.001, compared with the MPS group. ^#^*p* < 0.05, ^##^*p* < 0.01, ^###^*p* < 0.001, compared with the Dex group.

Micro-CT and angiography in each group were used to perform microvascular imaging of the femoral head (Figure 2A). The microvessel number, microvessel percentage, and microvessel volume were markedly improved in the CD+hUCMSC group in comparison to the MPS group (Figure 2B–2D). The implantation of hUCMSCs promoted microangiogenesis in the femoral head of the ONFH model rabbits.

#### hUCMSCs reduced the incidence of empty lacunae and increased the number of CD31+ microvessels

**Figure 3:**
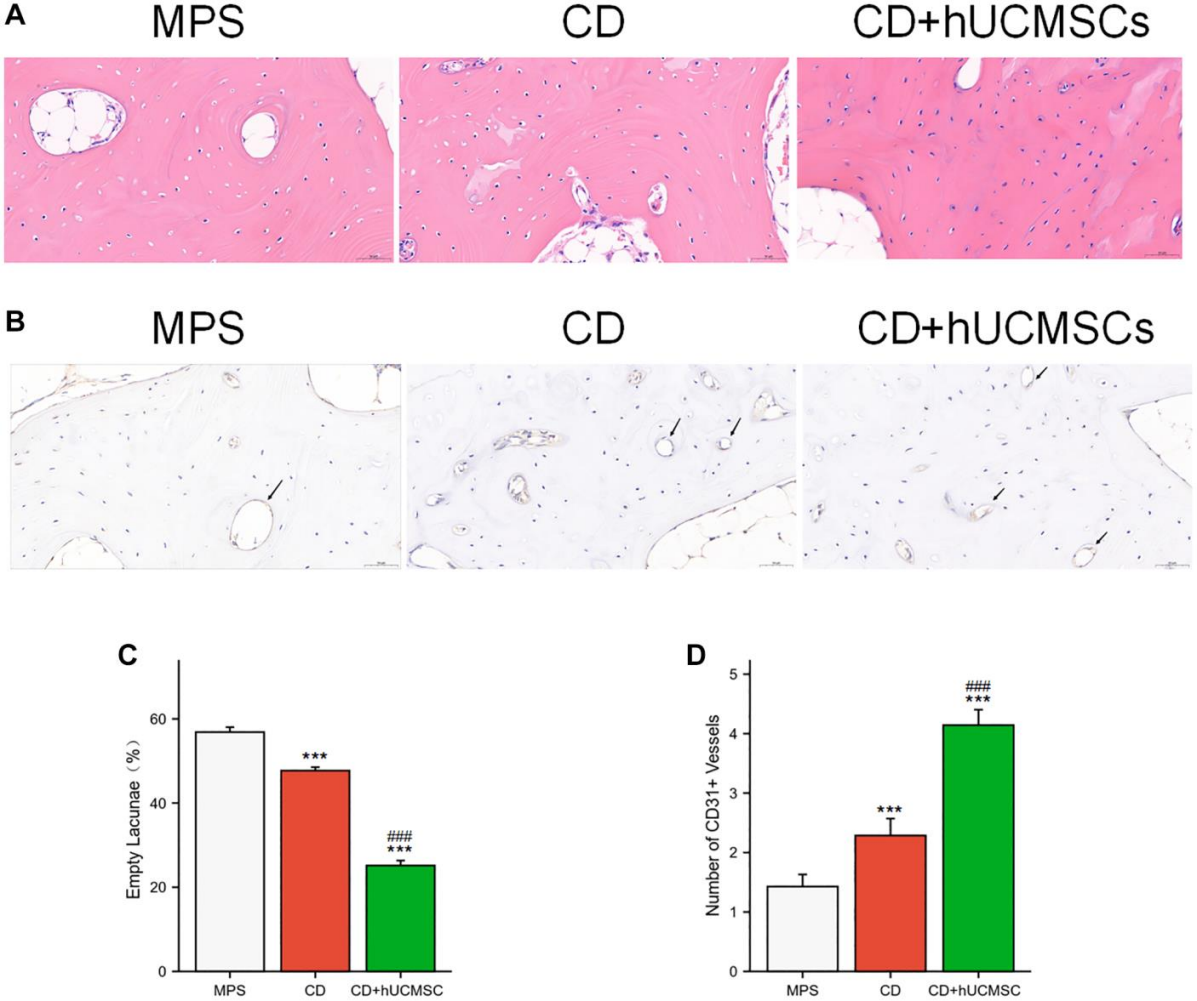
**hUCMSCs reduced the incidence of empty lacunae and increased the number of CD31+ microvessels.** (**A**) The bone tissues were analyzed via HE staining. (**B**) Immunohistochemistry staining of CD31. (**C**) Quantitative analysis of empty lacunae. (**D**) Quantitative analysis of the number of CD31+ vessels. The data are presented as the means ± SD (*n* = 7). ^*^p < 0.05, ^**^p < 0.01, ^***^p < 0.001, compared with the MPS group. ^#^*p* < 0.05, ^##^*p* < 0.01, ^###^*p* < 0.001, compared with the Dex group.

Osteonecrosis and angiogenesis were evaluated using HE staining and immunohistochemical CD31 staining. In the MPS group, many empty lacunae (formerly occupied by cells) and nuclear pyknosis (shrinkage of cell nuclei) were observed. Conversely, the CD+hUCMSC group exhibited fewer empty lacunae (Figure 3A). Implantation of hUCMSCs resulted in a remarkable reduction in the occurrence of empty lacunae in the femoral head of the ONFH rabbit model (Figure 3C). Furthermore, the CD+hUCMSC group exhibited a significantly higher number of CD31+ microvessels compared with the MPS group (Figure 3B, 3D). The implantation of hUCMSCs reduced the occurrence of empty lacunae in the femoral head of the ONFH rabbit model and promoted the generation of CD31+ microvessels.

### *In vitro* research

#### scRNA-seq analyzed and identified different cell types and ECs clusters

**Figure 4:**
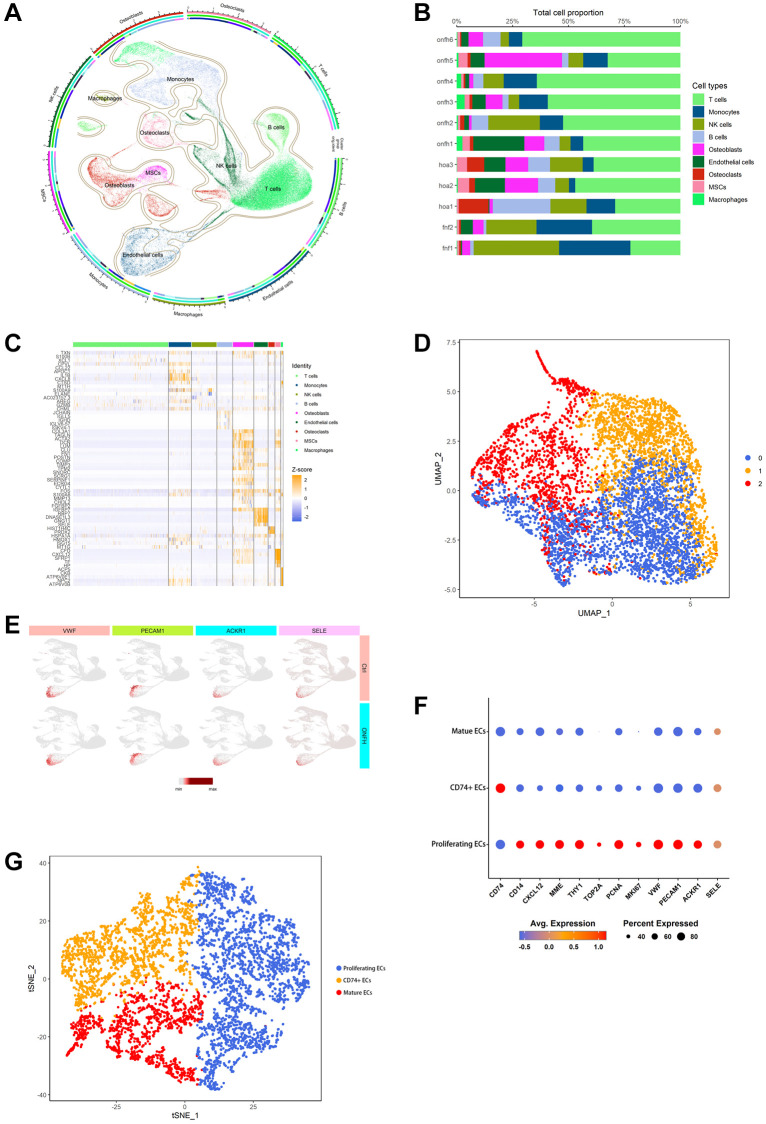
**scRNA-seq was used to analyze and identify different cell types and EC clusters.** (**A**) Cell type annotation of 9 clusters. (**B**) The percentage of clusters and cell types in each sample was represented on a proportion chart. (**C**) The heat map shows the top 10 marker genes in each cluster. (**D**) Two-dimensional plots of UMAP dimensionality reduction of the ECs. (**E**) ECs signature genes embedded on t-SNE dimension reduction map. (**F**) Dot plot showing marker genes for ECs subtypes. (**G**) tSNE shows the color-coded clustering for ECs.

We identified and categorized each cluster of cell types (Figure 4A), as follows: T cells, Monocytes, NK cells, B cells, Osteoblasts, endothelial cells (ECs), Osteoclasts, MSCs, and Macrophages. The proportion of each group of cells in the sample is shown in Figure 4B. The heat map shows the top 10 marker genes in each cluster (Figure 4C). Figure 4D shows ECs were clustered into 3 subtypes, while Figure 4E shows signature genes embedded on t-SNE dimension reduction map. Figure 4F presents a dot plot showing marker genes for ECs subtypes. The ECs were divided into three subtypes based on their proliferating capacity and the extent of their cell development. Three possible subtypes were identified: (0) proliferating ECs, (1) CD74+ ECs and (2) mature ECs (Figure 4G).

#### scRNA-seq analysis of the inter-cellular communication and DEGs in ECs

**Figure 5:**
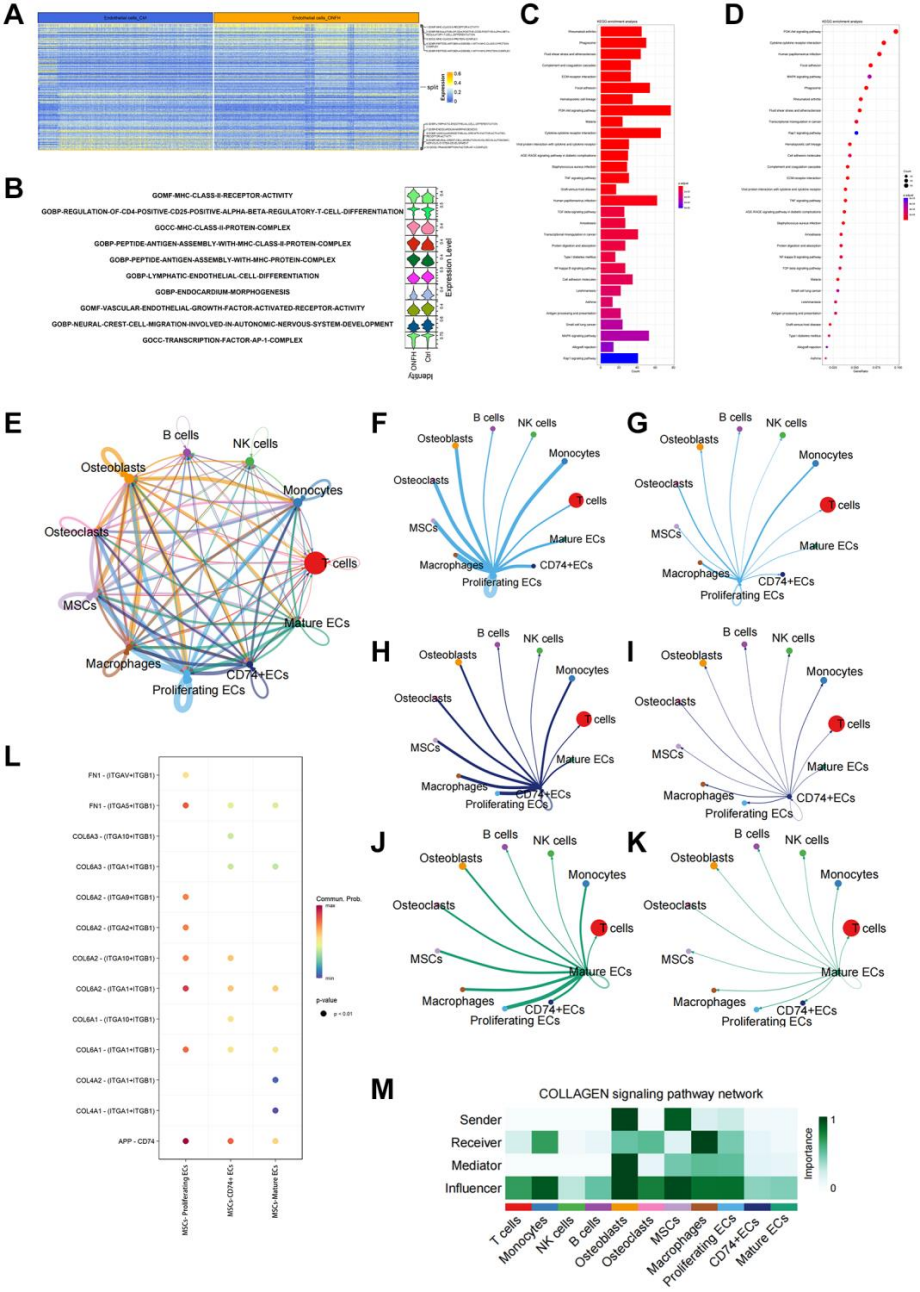
**scRNA-seq analysis of the inter-cellular communication and DEGs in ECs.** (**A**, **B**) GO enrichment analysis of DEGs in ECs. Heat map and violin plot showing top 5 up- or down-regulated signaling pathways in ECs. (**C**, **D**) KEGG enrichment analysis of DEGs in ECs. (**E**) Capacity for inter-cellular communication between ECs and other cells. (**F**, **G**) The counts and weights of ligand receptors among proliferating ECs and other cells. (**H**, **I**) The counts and weights of ligand receptors among CD74+ ECs and other cells. (**J**, **K**) The counts and weights of ligand receptors among mature ECs and other cells. (**L**) Ligand-receptor interactions between MSCs and ECs. (**M**) Inter-cellular communication in Collagen signaling pathway network.

We analyzed five significantly up, or down-regulated signaling pathways in ECs in the control and ONFH groups (Figure 5A, 5B). It was revealed that the VEGF signaling pathway was markedly down-regulated in the ONFH group. Additionally, we performed KEGG enrichment analysis of DEGs in ECs (Figure 5C, 5D), and PI3K/AKT signaling pathway expression was found to be markedly down-regulated in the ONFH group. This result suggests that the VEGF and PI3K/AKT signaling pathways are crucial in ONFH pathogenesis. Next, we analyzed the inter-cellular communication between ECs and other cells (Figure 5E–5M). We found that MSCs have strong inter-cellular communication with proliferating ECs via “COL6A2-ITGA1/ITGB1”, and the collagen pathway signaling network also suggested that MSCs affect proliferating ECs via the collagen pathway. Cell migration and angiogenesis are linked to the integrin α1β1, which is coded by the ITGA1 and ITGB1 genes. Furthermore, the activation of the PI3K/AKT signaling pathway downstream plays a crucial role in regulating cell survival and migration. This factor suggests that the effect of MSCs on ECs may be achieved through the “COL6A2-ITGA1/ITGB1” pathway. This hypothesis was subsequently tested using *in vitro* experiments.

#### Identification of the BMECs and the effect of Dex on the viability of BMECs

**Figure 6:**
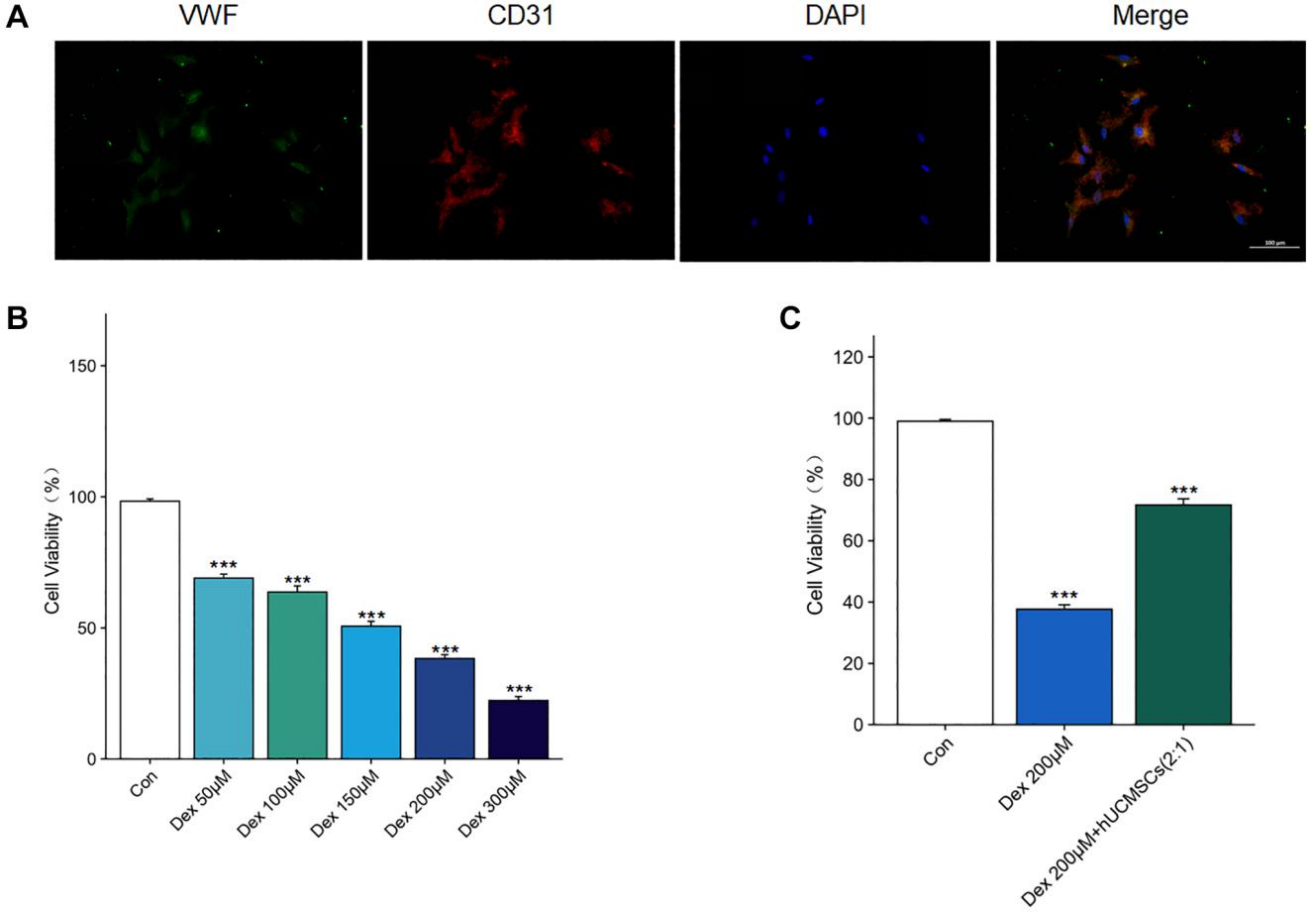
**Identification of the BMECs and the effect of Dex on the viability of BMECs.** (**A**) The immunofluorescence staining of CD31 and VWF was used to identify BMECs. (**B**) The effects of Dex (50, 100, 150, 200, 300 μM) on the cell viability were determined by CCK-8 assay. (**C**) The effects of hUCMSCs on the cell viability under 200 μM Dex stimulation were determined by CCK-8 assay. The data are presented as the means ± SD (*n* = 3). ^*^p < 0.05, ^**^p < 0.01, ^***^p < 0.001, compared with the Con group.

Primary human BMECs were extracted to understand the mechanism of action of hUCMSCs on steroid-induced ONFH. Under light microscopy, the cells were shown to be spindle-shaped or polygonal, and when the cells grew confluent, they presented a cobblestone morphology. Furthermore, angiogenic assays demonstrated the biological properties of primary cells for angiogenesis. The cells co-expressed CD31 and VWF by immunofluorescence detection, suggesting that the primary cells were BMECs (Figure 6A).

The Dex treatment of BMECs for 24 h was detected using CCK-8. We found that Dex inhibited BMECs’ viability in a concentration-dependent manner. Moreover, when the Dex concentration was 200 μM (Figure 6B), the cell viability of Dex-treated BMECs was significantly inhibited. However, the inhibitory effect of Dex was reversed after co-culture with hUCMSCs (Figure 6C). Therefore, 200 μM Dex will be used for subsequent experiments.

#### hUCMSCs improved the migration ability and angioplasty of Dex-treated BMECs

**Figure 7:**
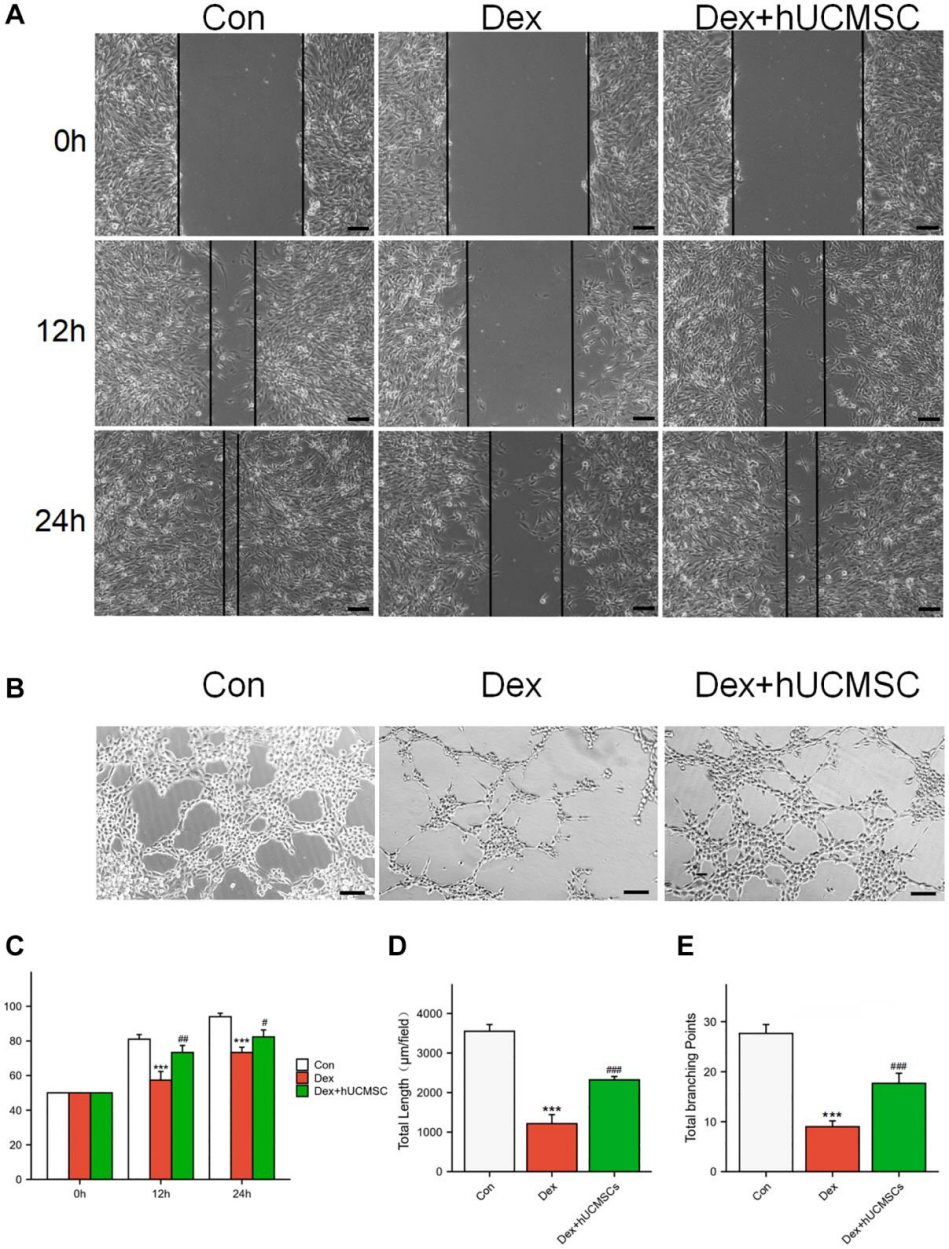
**hUCMSCs improved the migration ability and angioplasty of Dex-treated BMECs.** (**A**) Wound healing at 0, 12, and 24 h after Dex treatment. (**B**) Tube formation assay at 12 h after Dex treatment. (**C**) Scratch closure rate in three groups. (**D**, **E**) Total length and Total branching points in three groups. The data are presented as the means ± SD (*n* = 3). ^*^p < 0.05, ^**^p < 0.01, ^***^p < 0.001, compared with the Con group. ^#^*p* < 0.05, ^##^*p* < 0.01, ^###^*p* < 0.001, compared with the Dex group. Scale bars: 50 μm.

To study the effects of hUCMSCs on the migration and angiogenic abilities of BMECs, we conducted wound healing and tube formation assays (Figure 7A, 7B). In the Dex group, Dex significantly inhibited the migration ability of BMECs compared with the Con group. In the Dex+hUCMSC group, the impaired migration ability of BMECs was reversed after co-culture with hUCMSCs (Figure 7C). In tube formation assays, Dex-treated BMECs had shorter tubes and fewer branching points, and the angiogenic ability of BMECs was enhanced after co-culture with hUCMSCs (Figure 7D, 7E).

#### hUCMSCs played a protective role by regulating FAK/PI3K/AKT signaling pathway through COL6A2

**Figure 8:**
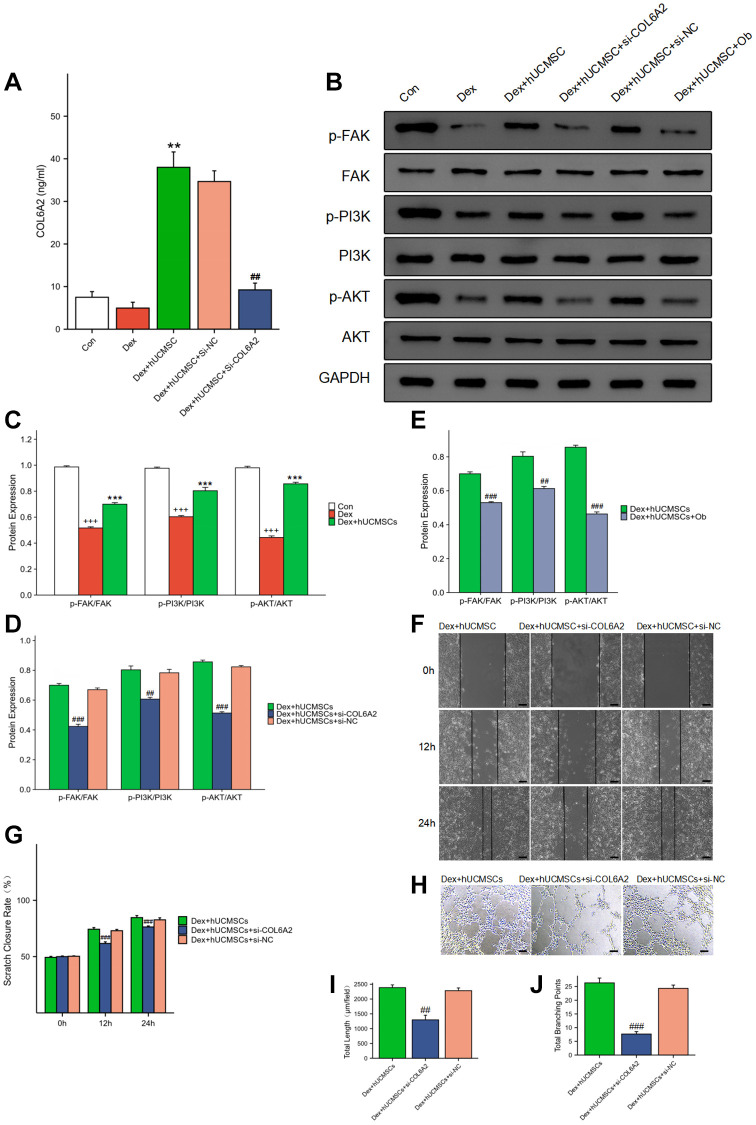
**hUCMSCs played a protective role by regulating FAK/PI3K/AKT signaling pathway through COL6A2.** (**A**) COL6A2 levels in the cell supernatants, assessed via ELISA. (**B**–**E**) The protein level of p-FAK, FAK, p-PI3K, PI3K, p-AKT, AKT and GAPDH in BMECs by Western blot. (**F**) Wound healing assay at 0, 12, and 24 h after co-culture with hUCMSCs. (**G**) Scratch closure rate in three groups. (**H**) Tube formation assay at 12 h after co-culture with hUCMSCs. (**I**, **J**) Total length and Total branching points in three groups. The data are presented as the means ± SD (*n* = 3). ^+^*p* < 0.05, ^++^*p* < 0.01, ^+++^*p* < 0.001, compared with the Con group. ^*^p < 0.05, ^**^p < 0.01, ^***^p < 0.001, compared with the Dex group. ^#^*p* < 0.05, ^##^*p* < 0.01, ^###^*p* < 0.001, compared with the Dex+hUCMSC group. Scale bars: 50 μm.

**Figure 9:**
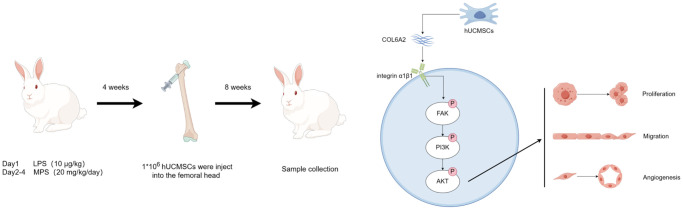
hUCMSCs promote steroid-induced ONFH repair by improving BMECs function.

Using ELISA analysis, we discovered that the concentration of COL6A2 was significantly higher in the culture supernatant of the Dex+hUCMSC group compared with that in the Con and Dex groups (Figure 8A). Furthermore, the level of p-FAK/FAK, p-PI3K/PI3K, and p-AKT/AKT was markedly increased in BMECs in the Dex+hUCMSC group compared with the Dex group (Figure 8B, 8C). To verify the role of COL6A2, we knocked down COL6A2 in hUCMSCs, and the results demonstrated that COL6A2 knockdown reversed the up-regulation of p-FAK/FAK, p-PI3K/PI3K, and p-AKT/AKT levels (Figure 8D). It was suggested that COL6A2 activated FAK/PI3K/AKT. Next, to verify whether the action of COL6A2 was mediated through integrin α1β1, we used Obtustatin (Ob) as a selective integrin α1β1 blocker [21]. The results demonstrated that p-FAK/FAK, p-PI3K/PI3K, and p-AKT/AKT levels were markedly down-regulated after Ob treatment (Figure 8E). It is suggested that the activation of the FAK/PI3K/AKT signaling pathway by COL6A2 is mediated through integrin α1β1. Furthermore, COL6A2 knockdown reversed the protective effect of hUCMSCs in wound healing and tube formation assay (Figure 8F–8J). These results suggested that hUCMSCs can promote the migration and angiogenesis of Dex-treated BMECs *in vitro*, and the mechanism of action is that hUCMSCs secrete COL6A2 to activate FAK/PI3K/AKT signaling pathway through integrin α1β1 (Figure 9).

## DISCUSSION

Glucocorticoids are widely used to treat autoimmune diseases and allergic diseases. However, long-term high-dose of such medication may cause steroid-induced ONFH. Glucocorticoids are one of the primary risk factors in nontraumatic ONFH [22]. The pathogenesis of steroid-induced ONFH remains unclear, although the vascular injury hypothesis [23] is one of the most prominent mechanisms. The vascular injury hypothesis suggests that ECs injury-related vascular injury reduces or interrupts blood flow to the femoral head and ultimately leads to the onset of ONFH. The BMECs play an essential role in vascular homeostasis and angiogenesis in bone. BMECs primarily consist of H-type and L-type microvascular ECs [4], of which H-type ECs are primarily distributed in the metaphysis and can promote bone repair and regeneration [24]. Simultaneously, the damage and dysfunction of BMECs are crucial in the development of steroid-induced ONFH. Glucocorticoids can directly damage ECs and lead to the development of a hypercoagulable state [25]. Therefore, the protection and rescue of BMECs function represent one of the possible approaches to prevent and treat steroid-induced ONFH.

Recently, a significant number of studies have demonstrated that hUCMSCs and their exosomes have powerful functions in promoting angiogenesis and injury repair [26]. Their mechanisms of action may include promoting cytokine secretion via ECs, inhibiting apoptosis, and regulating ECs’ oxidative stress injury. It was found that hUCMSCs can promote VEGF secretion by ECs through their exosomes, accelerate ECs proliferation and migration, and promote angiogenesis [27]. In a diabetic model, hUCMSCs and their exosomes can protect ECs and promote diabetic wound healing by regulating ECs’ oxidative stress injury mediated by high glucose [28].

CD is one operative method of treating ONFH and is applied among patients in the early stages before the femoral head has yet to collapse. The main objective of CD is to remove sclerotic dead bone within the lesion, reduce intraosseous pressure and promote angiogenesis near the decompression channel, ultimately delaying the disease’s progression or curing the disease [29]. However, the results of CD treatment alone are variable, and their effectiveness has been challenged [30]. To obtain better efficacy, cell therapy combined with CD was proposed and has achieved better results [31–33]. This result suggests that the approach of cell therapy combined with CD treatment may be a promising direction for the future. However, the application of autologous-derived MSCs has some drawbacks. For example, the acquisition of MSCs is traumatic, the number of MSCs acquired is limited, and treatment efficacy is affected by the age of the patient [34, 35]. Moreover, the advantages of hUCMSCs, such as their low immunogenicity, simple acquisition, and ethical compliance, make hUCMSCs more suitable for cell therapy applications. Additionally, using this method, cells are delivered directly into the lesion through the CD channel to exert their repairing effect, thereby avoiding stem cell homing. In *in vivo* experiment, we found that hUCMSCs improved femoral microcirculation, elevated the number of CD31+ microvessels, and alleviated the occurrence of osteonecrosis in a rabbit model of steroid-induced ONFH. It is suggested that hUCMSCs prevent the occurrence of steroid-induced ONFH by improving bone microcirculation.

Collagen VI primarily exists in the extracellular matrix. In addition to its anchoring role, Collagen VI can also regulate apoptosis and oxidative stress and promote cell growth [36]. Moreover, Collagen VI consists of three primary isoforms encoded by three genes, COL6A1, COL6A2, and COL6A3, respectively. Collagen VI can bind to many cell surface receptors to perform biological functions, including integrin α1β1 [37]. Integrins are cell surface transmembrane receptors, and activated integrins can induce changes in intracellular signaling pathways, which primarily include the FAK/PI3K/AKT signaling pathway [38]. The FAK is a non-receptor tyrosine kinase that can be activated by VEGF and integrins [39], and it is involved in ECs proliferation, survival, and migration [40, 41]. Therefore, FAK plays a pivotal role in angiogenesis [42]. We found that the migration and angiogenic ability of BMECs received inhibition after Dex treatment, while p-FAK/FAK, p-PI3K/PI3K, and p-AKT/AKT expression were reduced. After co-culture with hUCMSCs, the inhibition of BMECs migration and angiogenic ability was reversed, while p-FAK/FAK, p-PI3K/PI3K, and p-AKT/AKT levels were up-regulated. The supernatant of the hUCMSCs culture medium had a markedly higher concentration of COL6A2 than that in the Con group, and the knockdown of COL6A2 in hUCMSCs reversed the levels of p-FAK/FAK, p-PI3K/PI3K, and p-AKT/AKT. It is suggested that hUCMSCs may apply their protective effects on BMECs by activating the FAK/PI3K/AKT signaling pathway through COL6A2. Concurrently, the up-regulation of p-FAK/FAK, p-PI3K/PI3K, and p-AKT/AKT levels were reversed after the use of the specific inhibitor of integrin α1β1, Ob, which suggests that FAK/PI3K/AKT signaling pathway activation by COL6A2 is mediated through integrin α1β1.

## CONCLUSION

In conclusion, this study revealed that hUCMSCs can promote femoral head angiogenesis and bone repair in the Steroid-induced ONFH rabbit model. Moreover, the hUCMSCs can promote the migration and angiogenesis of Dex-treated BMECs *in vitro*, and the mechanism of action is that hUCMSCs secrete COL6A2 to activate FAK/PI3K/AKT signaling pathway through integrin α1β1.

## Supplementary Materials

Supplementary Figure 1

## References

[1] Zhao DW, Yu M, Hu K, Wang W, Yang L, Wang BJ, Gao XH, Guo YM, Xu YQ, Wei YS, Tian SM, Yang F, Wang N, et al. Prevalence of Nontraumatic Osteonecrosis of the Femoral Head and its Associated Risk Factors in the Chinese Population: Results from a Nationally Representative Survey. Chin Med J (Engl). 2015; 128:2843–50. 10.4103/0366-6999.168017 26521779 PMC4756878

[2] Liao Y, Zhang P, Yuan B, Li L, Bao S. Pravastatin Protects Against Avascular Necrosis of Femoral Head via Autophagy. Front Physiol. 2018; 9:307. 10.3389/fphys.2018.00307 29686621 PMC5900057

[3] Riddle RC, Khatri R, Schipani E, Clemens TL. Role of hypoxia-inducible factor-1alpha in angiogenic-osteogenic coupling. J Mol Med (Berl). 2009; 87:583–90. 10.1007/s00109-009-0477-9 19415227 PMC3189695

[4] Kusumbe AP, Ramasamy SK, Adams RH. Coupling of angiogenesis and osteogenesis by a specific vessel subtype in bone. Nature. 2014; 507:323–8. 10.1038/nature13145 24646994 PMC4943525

[5] Zhang Q, L V J, Jin L. Role of coagulopathy in glucocorticoid-induced osteonecrosis of the femoral head. J Int Med Res. 2018; 46:2141–8. 10.1177/0300060517700299 28459353 PMC6023042

[6] Kerachian MA, Séguin C, Harvey EJ. Glucocorticoids in osteonecrosis of the femoral head: a new understanding of the mechanisms of action. J Steroid Biochem Mol Biol. 2009; 114:121–8. 10.1016/j.jsbmb.2009.02.007 19429441 PMC7126235

[7] Butler J, Epstein SE, Greene SJ, Quyyumi AA, Sikora S, Kim RJ, Anderson AS, Wilcox JE, Tankovich NI, Lipinski MJ, Ko YA, Margulies KB, Cole RT, et al. Intravenous Allogeneic Mesenchymal Stem Cells for Nonischemic Cardiomyopathy: Safety and Efficacy Results of a Phase II-A Randomized Trial. Circ Res. 2017; 120:332–40. 10.1161/CIRCRESAHA.116.309717 27856497

[8] Lanzoni G, Linetsky E, Correa D, Messinger Cayetano S, Alvarez RA, Kouroupis D, Alvarez Gil A, Poggioli R, Ruiz P, Marttos AC, Hirani K, Bell CA, Kusack H, et al. Umbilical cord mesenchymal stem cells for COVID-19 acute respiratory distress syndrome: A double-blind, phase 1/2a, randomized controlled trial. Stem Cells Transl Med. 2021; 10:660–73. 10.1002/sctm.20-0472 33400390 PMC8046040

[9] Álvaro-Gracia JM, Jover JA, García-Vicuña R, Carreño L, Alonso A, Marsal S, Blanco F, Martínez-Taboada VM, Taylor P, Martín-Martín C, DelaRosa O, Tagarro I, Díaz-González F. Intravenous administration of expanded allogeneic adipose-derived mesenchymal stem cells in refractory rheumatoid arthritis (Cx611): results of a multicentre, dose escalation, randomised, single-blind, placebo-controlled phase Ib/IIa clinical trial. Ann Rheum Dis. 2017; 76:196–202. 10.1136/annrheumdis-2015-208918 27269294

[10] Vega A, Martín-Ferrero MA, Del Canto F, Alberca M, García V, Munar A, Orozco L, Soler R, Fuertes JJ, Huguet M, Sánchez A, García-Sancho J. Treatment of Knee Osteoarthritis With Allogeneic Bone Marrow Mesenchymal Stem Cells: A Randomized Controlled Trial. Transplantation. 2015; 99:1681–90. 10.1097/TP.0000000000000678 25822648

[11] Ahani-Nahayati M, Niazi V, Moradi A, Pourjabbar B, Roozafzoon R, Keshel SH, Baradaran-Rafii A. Umbilical Cord Mesenchymal Stem/Stromal Cells Potential to Treat Organ Disorders; An Emerging Strategy. Curr Stem Cell Res Ther. 2022; 17:126–46. 10.2174/1574888X16666210907164046 34493190

[12] Chen Y, Qian H, Zhu W, Zhang X, Yan Y, Ye S, Peng X, Li W, Xu W. Hepatocyte growth factor modification promotes the amelioration effects of human umbilical cord mesenchymal stem cells on rat acute kidney injury. Stem Cells Dev. 2011; 20:103–13. 10.1089/scd.2009.0495 20446811

[13] Sabapathy V, Sundaram B, V M S, Mankuzhy P, Kumar S. Human Wharton's Jelly Mesenchymal Stem Cells plasticity augments scar-free skin wound healing with hair growth. PLoS One. 2014; 9:e93726. 10.1371/journal.pone.0093726 24736473 PMC3988008

[14] Chen C, Qu Z, Yin X, Shang C, Ao Q, Gu Y, Liu Y. Efficacy of umbilical cord-derived mesenchymal stem cell-based therapy for osteonecrosis of the femoral head: A three-year follow-up study. Mol Med Rep. 2016; 14:4209–15. 10.3892/mmr.2016.5745 27634376 PMC5101965

[15] Tian G, Liu C, Gong Q, Yu Z, Wang H, Zhang D, Cong H. Human Umbilical Cord Mesenchymal Stem Cells Improve the Necrosis and Osteocyte Apoptosis in Glucocorticoid-Induced Osteonecrosis of the Femoral Head Model through Reducing the Macrophage Polarization. Int J Stem Cells. 2022; 15:195–202. 10.15283/ijsc21120 34965999 PMC9148830

[16] Zhao D, Zhang F, Wang B, Liu B, Li L, Kim SY, Goodman SB, Hernigou P, Cui Q, Lineaweaver WC, Xu J, Drescher WR, Qin L. Guidelines for clinical diagnosis and treatment of osteonecrosis of the femoral head in adults (2019 version). J Orthop Translat. 2020; 21:100–10. 10.1016/j.jot.2019.12.004 32309135 PMC7152793

[17] Diniz IM, Chen C, Xu X, Ansari S, Zadeh HH, Marques MM, Shi S, Moshaverinia A. Pluronic F-127 hydrogel as a promising scaffold for encapsulation of dental-derived mesenchymal stem cells. J Mater Sci Mater Med. 2015; 26:153. 10.1007/s10856-015-5493-4 25773231 PMC4477746

[18] Sun Y, Feng Y, Zhang C, Cheng X, Chen S, Ai Z, Zeng B. Beneficial effect of autologous transplantation of endothelial progenitor cells on steroid-induced femoral head osteonecrosis in rabbits. Cell Transplant. 2011; 20:233–43. 10.3727/096368910X522234 20719092

[19] Yu H, Yue J, Wang W, Liu P, Zuo W, Guo W, Zhang Q. Icariin promotes angiogenesis in glucocorticoid-induced osteonecrosis of femoral heads: In vitro and in vivo studies. J Cell Mol Med. 2019; 23:7320–30. 10.1111/jcmm.14589 31507078 PMC6815836

[20] Yao L, Hu X, Yuan M, Liu P, Zhang Q, Wang Z, Chen P, Xiong Z, Wu L, Dai K, Jiang Y. Human umbilical cord-derived mesenchymal stromal cells alleviate liver cirrhosis through the Hippo/YAP/Id1 pathway and macrophage-dependent mechanism. Int Immunopharmacol. 2023; 123:110456. 10.1016/j.intimp.2023.110456 37494836

[21] Moraes JA, Frony AC, Dias AM, Renovato-Martins M, Rodrigues G, Marcinkiewicz C, Assreuy J, Barja-Fidalgo C. Data in support of alpha1beta1 and integrin-linked kinase interact and modulate angiotensin II effects in vascular smooth muscle cells. Data Brief. 2015; 6:330–40. 10.1016/j.dib.2015.11.053 26862579 PMC4706604

[22] Assouline-Dayan Y, Chang C, Greenspan A, Shoenfeld Y, Gershwin ME. Pathogenesis and natural history of osteonecrosis. Semin Arthritis Rheum. 2002; 32:94–124. 12430099

[23] Kerachian MA, Harvey EJ, Cournoyer D, Chow TY, Séguin C. Avascular necrosis of the femoral head: vascular hypotheses. Endothelium. 2006; 13:237–44. 10.1080/10623320600904211 16990180

[24] Zhang J, Pan J, Jing W. Motivating role of type H vessels in bone regeneration. Cell Prolif. 2020; 53:e12874. 10.1111/cpr.12874 33448495 PMC7507571

[25] Boss JH, Misselevich I. Osteonecrosis of the femoral head of laboratory animals: the lessons learned from a comparative study of osteonecrosis in man and experimental animals. Vet Pathol. 2003; 40:345–54. 10.1354/vp.40-4-345 12824505

[26] Hu Z, Jiang Z, Meng S, Liu R, Yang K. Research Progress on the Osteogenesis-Related Regulatory Mechanisms of Human Umbilical Cord Mesenchymal Stem Cells. Stem Cell Rev Rep. 2023; 19:1252–67. 10.1007/s12015-023-10521-5 36917312

[27] Qu Q, Pang Y, Zhang C, Liu L, Bi Y. Exosomes derived from human umbilical cord mesenchymal stem cells inhibit vein graft intimal hyperplasia and accelerate reendothelialization by enhancing endothelial function. Stem Cell Res Ther. 2020; 11:133. 10.1186/s13287-020-01639-1 32293542 PMC7092460

[28] Yan C, Xv Y, Lin Z, Endo Y, Xue H, Hu Y, Hu L, Chen L, Cao F, Zhou W, Zhang P, Liu G. Human Umbilical Cord Mesenchymal Stem Cell-Derived Exosomes Accelerate Diabetic Wound Healing via Ameliorating Oxidative Stress and Promoting Angiogenesis. Front Bioeng Biotechnol. 2022; 10:829868. 10.3389/fbioe.2022.829868 35174145 PMC8841645

[29] Mont MA, Jones LC, Hungerford DS. Nontraumatic osteonecrosis of the femoral head: ten years later. J Bone Joint Surg Am. 2006; 88:1117–32. 10.2106/JBJS.E.01041 16651589

[30] Hines JT, Jo WL, Cui Q, Mont MA, Koo KH, Cheng EY, Goodman SB, Ha YC, Hernigou P, Jones LC, Kim SY, Sakai T, Sugano N, et al. Osteonecrosis of the Femoral Head: an Updated Review of ARCO on Pathogenesis, Staging and Treatment. J Korean Med Sci. 2021; 36:e177. 10.3346/jkms.2021.36.e177 34155839 PMC8216992

[31] Hernigou P, Dubory A, Homma Y, Guissou I, Flouzat Lachaniette CH, Chevallier N, Rouard H. Cell therapy versus simultaneous contralateral decompression in symptomatic corticosteroid osteonecrosis: a thirty year follow-up prospective randomized study of one hundred and twenty five adult patients. Int Orthop. 2018; 42:1639–49. 10.1007/s00264-018-3941-8 29744647

[32] Kang JS, Suh YJ, Moon KH, Park JS, Roh TH, Park MH, Ryu DJ. Clinical efficiency of bone marrow mesenchymal stem cell implantation for osteonecrosis of the femoral head: a matched pair control study with simple core decompression. Stem Cell Res Ther. 2018; 9:274. 10.1186/s13287-018-1030-y 30359323 PMC6202854

[33] Li X, Xu X, Wu W. Comparison of bone marrow mesenchymal stem cells and core decompression in treatment of osteonecrosis of the femoral head: a meta-analysis. Int J Clin Exp Pathol. 2014; 7:5024–30. 25197374 PMC4152064

[34] Mantripragada VP, Boehm C, Bova W, Briskin I, Piuzzi NS, Muschler GF. Patient Age and Cell Concentration Influence Prevalence and Concentration of Progenitors in Bone Marrow Aspirates: An Analysis of 436 Patients. J Bone Joint Surg Am. 2021; 103:1628–36. 10.2106/JBJS.20.02055 33844657

[35] Lavrentieva A, Hoffmann A, Lee-Thedieck C. Limited Potential or Unfavorable Manipulations? Strategies Toward Efficient Mesenchymal Stem/Stromal Cell Applications. Front Cell Dev Biol. 2020; 8:316. 10.3389/fcell.2020.00316 32509777 PMC7248306

[36] Lamandé SR, Bateman JF. Collagen VI disorders: Insights on form and function in the extracellular matrix and beyond. Matrix Biol. 2018; 71-72:348–67. 10.1016/j.matbio.2017.12.008 29277723

[37] Pfaff M, Aumailley M, Specks U, Knolle J, Zerwes HG, Timpl R. Integrin and Arg-Gly-Asp dependence of cell adhesion to the native and unfolded triple helix of collagen type VI. Exp Cell Res. 1993; 206:167–76. 10.1006/excr.1993.1134 8387021

[38] Legate KR, Wickström SA, Fässler R. Genetic and cell biological analysis of integrin outside-in signaling. Genes Dev. 2009; 23:397–418. 10.1101/gad.1758709 19240129

[39] Chen XL, Nam JO, Jean C, Lawson C, Walsh CT, Goka E, Lim ST, Tomar A, Tancioni I, Uryu S, Guan JL, Acevedo LM, Weis SM, et al. VEGF-induced vascular permeability is mediated by FAK. Dev Cell. 2012; 22:146–57. 10.1016/j.devcel.2011.11.002 22264731 PMC3266538

[40] Dawson JC, Serrels A, Stupack DG, Schlaepfer DD, Frame MC. Targeting FAK in anticancer combination therapies. Nat Rev Cancer. 2021; 21:313–24. 10.1038/s41568-021-00340-6 33731845 PMC8276817

[41] Lechertier T, Hodivala-Dilke K. Focal adhesion kinase and tumour angiogenesis. J Pathol. 2012; 226:404–12. 10.1002/path.3018 21984450

[42] Zhao X, Guan JL. Focal adhesion kinase and its signaling pathways in cell migration and angiogenesis. Adv Drug Deliv Rev. 2011; 63:610–5. 10.1016/j.addr.2010.11.001 21118706 PMC3132829

