# Supplementary material for: Human umbilical cord mesenchymal stem cells promote steroid-induced osteonecrosis of the femoral head repair by improving microvascular endothelial cell function
Source: Aging (Albany NY). 2024 Apr 29;16(9):7928–45. doi: 10.18632/aging.205794 (PMC11132024; doi:10.18632/aging.205794)
Supplement: Supplementary Figure 1 [file aging-16-205794-s001.pdf]

SUPPLEMENTARY FIGURE

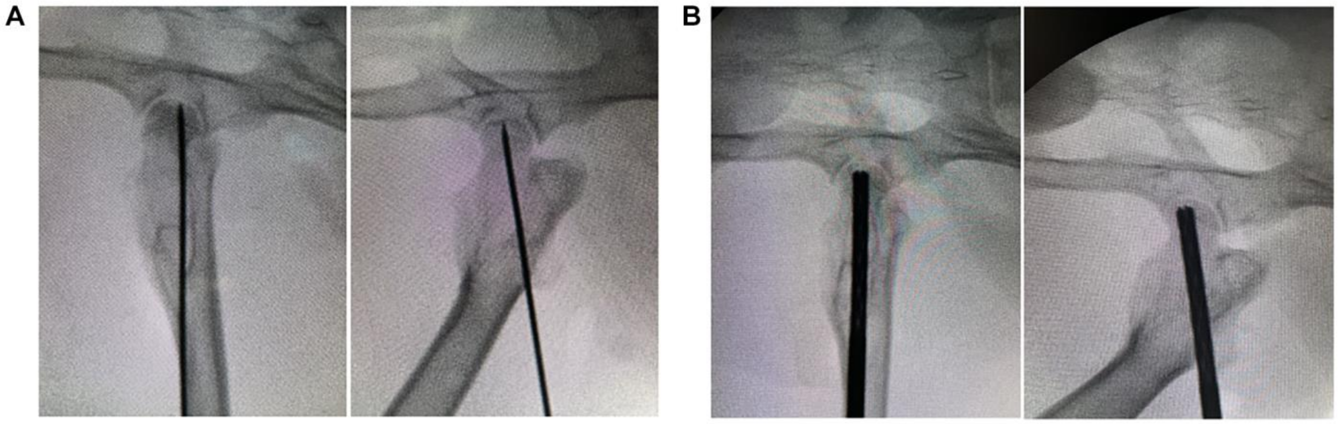

Supplementary Figure 1. X-ray during the animal surgery. (**A**) Kirschner wire was drilled along the center of the femoral neck; (**B**) Hollow drill was drilled into the femoral head.
